# Association between ultra-processed food consumption and gut microbiota in senior subjects with overweight/obesity and metabolic syndrome

**DOI:** 10.3389/fnut.2022.976547

**Published:** 2022-10-10

**Authors:** Alessandro Atzeni, María Ágeles Martínez, Nancy Babio, Prokopis Konstanti, Francisco J. Tinahones, Jesús Vioque, Dolores Corella, Montserrat Fitó, Josep Vidal, Isabel Moreno-Indias, Salvador Pertusa-Martinez, Andrea Álvarez-Sala, Olga Castañer, Albert Goday, Miguel Damas-Fuentes, Clara Belzer, Miguel Á. Martínez-Gonzalez, Frank B. Hu, Jordi Salas-Salvadó

**Affiliations:** ^1^Institut d'Investigació Sanitària Pere Virgili (IISPV), Reus, Spain; ^2^Universitat Rovira i Virgili, Department de Bioquímica i Biotecnologia, Unitat de Nutrició, Reus, Spain; ^3^Centro de Investigación Biomédica en Red Fisiopatología de la Obesidad y la Nutrición (CIBEROBN), Institute of Health Carlos III, Madrid, Spain; ^4^Laboratory of Microbiology, Wageningen University, Wageningen, Netherlands; ^5^Department of Endocrinology and Nutrition, Instituto de Investigación Biomédica de Málaga – IBIMA, Hospital Universitario Virgen de la Vic, Málaga, Spain; ^6^CIBER de Epidemiología y Salud Pública (CIBERESP), Instituto de Salud Carlos III, Madrid, Spain; ^7^Instituto de Investigación Sanitaria y Biomédica de Alicante, Universidad Miguel Hernández (ISABIAL-UMH), Alicante, Spain; ^8^Department of Preventive Medicine, University of Valencia, Valencia, Spain; ^9^Unit of Cardiovascular Risk and Nutrition, Institut Hospital del Mar de Investigaciones Médicas Municipal d‘Investigació Médica (IMIM), Barcelona, Spain; ^10^CIBER Diabetes y Enfermedades Metabólicas (CIBERDEM), Instituto de Salud Carlos III (ISCIII), Madrid, Spain; ^11^Department of Endocrinology, Institut d‘Investigacions Biomédiques August Pi Sunyer (IDIBAPS), Hospital Clinic, University of Barcelona, Barcelona, Spain; ^12^Centro Salud Cabo Huertas, Alicante, Spain; ^13^Medicine Department, Universitat Autònoma de Barcelona, Barcelona, Spain; ^14^IMIM, Hospital del Mar Medical Research Institute, Barcelona, Spain; ^15^Department of Preventive Medicine and Public Health, University of Navarra, Pamplona, Spain; ^16^Department of Nutrition, Harvard T. H. Chan School of Public Health, Boston, MA, United States; ^17^Channing Division for Network Medicine, Department of Medicine, Brigham and Women's Hospital and Harvard Medical School, Boston, MA, United States

**Keywords:** ultra-processed food, gut microbiota, mediterranean diet, 16s sequencing, overweight, obesity, metabolic syndrome

## Abstract

The production and consumption of ultra-processed foods (UPF) has increased considerably during the last years worldwide. Collective evidence shows the association between UPF consumption and adverse health outcomes, including inflammatory gastro-intestinal disorders and obesity. The gut microbiota has been suggested as potential mediator of the effects of UPF consumption on metabolism and health. However, few studies have been conducted in order to elucidate these aspects. Therefore, the aim of the present study was to assess the cross-sectional associations between UPF consumption and gut microbiota in a population of senior subjects (*n* = 645) within the frame of the PREDIMED-Plus trial. Eligible participants were men and women (aged 55–75 years), without documented history of cardiovascular disease at enrollment, with overweight/obesity (body mass index ≤ 27 and <40 kg/m^2^) and metabolic syndrome. Using the information of food frequency questionnaires, the consumption of UPF, expressed as a percentage of total dietary energy intake in kcal/day, was calculated considering those food items classified in group 4 of NOVA system. Population was categorized according to tertiles of UPF consumption. Taxonomic fecal microbiota information, along with blood biochemical parameters, anthropometric measurements and clinical data were obtained. Bioinformatics analysis was performed to study the association between fecal microbiota composition and UPF consumption. We observed that subjects allocated in the highest tertile of UPF consumption (21.4 ± 5.0 % kcal/day) presented lower adherence to MedDiet (*p* < 0.001) and higher total energy intake (*p* < 0.001). The taxonomic analysis of the fecal microbiota revealed a significant (Benjamini-Hochberg adjusted *p* < 0.2) positive association between specific taxa and tertiles (T) of UPF consumption: *Alloprevotella* (*p* = 0.041 vs. T2; *p* = 0.065 vs. T3), *Negativibacillus* (*p* = 0.096 vs. T3), *Prevotella* (*p* = 0.116 vs. T3), and *Sutterella* (*p* = 0.116 vs. T2). UPF consumption was positively associated with lower adherence to MedDiet and higher total energy intake in senior subjects with overweight obesity and metabolic syndrome. In addition, positive association with specific fecal microbiota taxa related to inflammatory gastro-intestinal diseases and low consumption of fruits and vegetables, was observed.

## Introduction

Production and consumption of ultra-processed food (UPF) has increased considerably during the last years worldwide, displacing foods that are usually consumed in healthy eating patterns, abundant in unprocessed vegetables, fruits, legumes and nuts among others (1, 2).

UPF are defined as multi-ingredient industrial formulations manufactured from substances derived from foods and additives or ingredients not normally used in home food preparation (such as hydrogenated or unesterified oils, protein isolate, maltodextrin, casein, and gluten), retaining little or none of their original food characteristics. UPF include food substances not used in culinary preparations, in particular flavorings, colorings, sweeteners, emulsifiers and other additives used to mimic the sensory qualities of unprocessed or to mask undesirable qualities of the final product (3, 4). They are frequently highly palatable and easy to eat, as they usually can be consumed at the time of purchase or with very little preparation. As a result, the UPF are commonly rich in energy density, added sugars, salt, and saturated and trans fatty acids, and low in fiber, protein, and micronutrients. According to the NOVA food classification system (4, 5) foods and food products can be classified into four categories according to the degree of processing: low or unprocessed foods (i.e., fresh fruits, vegetables or legumes), culinary ingredients (i.e., honey or sugar), processed foods (fruit juices or canned vegetables with salt), and ultra-processed foods (such as pizza or biscuits).

A growing body of evidence shows an association between UPF consumption and an increased risk of chronic diseases such as obesity (5–7), metabolic syndrome (MetS) (5), diabetes (8, 9), dyslipidemia (10), cardiovascular disease (CVD) (5, 11), cancer (12), and all-cause mortality (13, 14). Most of the studies relating UPF consumption and health outcomes are cross-sectional or prospective epidemiologic studies with a relatively low level of scientific evidence of cause-effect relationship. However, some recent clinical studies indicate that the decrease in the amount of UPF consumed should help improve strategies for cardiovascular risk factors in patients in secondary care (14, 15). These findings should serve as an incentive for limiting consumption of UPF, and promote natural or minimally processed foods, as several national nutritional policies recommend (8, 16).

Considering the global increase in consumption of UPFs and the growing evidence linking its excessive consumption to disease outcomes, it is important to outline the possible mechanisms by which this type of food may have detrimental health effects. One of the suggested mechanisms by which UPF consumption may have potential effects on metabolism and health is through the modulation of gut microbiota composition and function (7, 17). In fact, there is growing evidence demonstrating that diet may have an effect on cardiovascular risk factors and metabolism through gut microbiota modulation. Food processing alters the provision of macronutrient substrate to the colon due to differing digestibility, and thereby may impact the microbiota and its metabolic activity (17). Other additives and nutrient constituents have also demonstrated to modulate the host gut microbiota after consumption (7, 18). However, few studies have been conducted in order to explore the relationship between UPF and gut microbiota, and therefore further investigation is required in this sense.

The aim of the present study was to assess the baseline cross-sectional associations between UPF consumption and gut microbiota in a population of senior subjects with overweight/obesity and MetS within the frame of the PREDIMED-Plus trial.

## Materials and methods

### Study design and participants

The present cross-sectional study was conducted within the frame of the PREDIMED (PREvención con DIeta MEDiterránea)-Plus clinical trial. This trial aims to assess the long-term effects of an intensive weight loss lifestyle intervention based on an energy-restricted Mediterranean diet (MedDiet), physical activity (PA) promotion, and behavioral support (intervention group) on CVD events and mortality, vs. a control group following an energy-unrestricted MedDiet without any advice to increase PA (19). The trial was registered at the International Standard Randomized Controlled Trial (Number: ISRCTN89898870 -data of registration: 2014-) and approved by the institutional review board of all participating institutions. A detailed protocol is available at the web page http://predimedplus.com/. Eligible participants were men and women (aged 55–75 years), without documented history of CVD at enrollment, with overweight/obesity (body mass index (BMI) ≤ 27 and <40 kg/m^2^) and who met at baseline at least three components of the MetS.

The present study encompasses a subsample of participants from the PREDIMED-Plus recruiting centers of Reus, Barcelona (IMIM), Alicante, and Valencia, with available fecal microbiota data at baseline (*n* = 656).

### Dietary assessments

Dietary intake was assessed using a validated 143 item semiquantitative food frequency questionnaires (FFQs) (20) administered by trained dietitians. Participants reported their average frequency and quantity of food consumed during the previous year. The intake of each item was calculated by multiplying a typical portion size by frequency of consumption (9 possible responses ranging from never to > 6 times/day). Spanish food composition tables (21) were used to derive nutrient (sodium, saturated and trans fatty acids), fiber, alcohol (g/day), and total energy intake (kcal/day), as well as to determine consumption of specific food groups, such as fruits and vegetables (g/day).

Food and beverage items of FFQ were classified in accordance with the NOVA system proposed by Monteiro et al. (22). Foods and drinks were allocated in four different groups according to the nature, extent and purpose of their industrial processing, considering a variety of physical, biological, and chemical methods, as well as use of additives. For the purposes of the current study, we considered food items classified in the NOVA group 4 (G4), including “ultra-processed food and drink products.” These items were further classified into subgroups: dairy products; processed meat; pre-prepared dishes, snacks and fast-foods; sweets; non-alcoholic beverages; alcoholic beverages (Table 1). Using the information of FFQs we calculated the consumption of UPF G4 and expressed as a percentage of total energy intake (in kcal/day). The proportion of UPF in the total diet was calculated for each participant and categorized in tertiles, corresponding to low (first tertile), medium (second tertile), and high (third tertile) consumption.

**Table 1:** List of food items from the PREDIMED-Plus food frequency questionnaires allocated in Group 4 (ultra-processed food and drink products) according to NOVA classification system.

Dairy products	Industrially-produced milkshakes
	Flavored Petit Suisse yogurt
	Custard, crème caramel flan, pudding
	Ice-cream
	Creamy cheese spreads
Processed meats	Sandwich (deli) ham
	Cured cold meats
	Pâté
	Burger
Sweets	Biscuits
	Whole meal biscuits
	Breakfast cereals
	Chocolate biscuits
	Industrially-produced confectionery
	Donuts
	Muffins, cupcakes
	Industrially-produced cakes
	Churros
	Chocolates
	Soluble cocoa powder
	Nougat
	Marzipan, shortbread biscuits
	Jam
Pre-prepared dishes,	Mustard
snacks and fast-foods	Mayonnaise
	Tomato sauce, Ketchup
	Margarine
	Packaged snacks
	Croquettes
	Instant soup
	Pizza
Non-alcoholic beverages	Soft drinks
	No-added sugar drinks
Alcoholic beverages	Liquors, anisette
	Whisky, gin, vodka, cognac

### General assessments, anthropometric and biochemical measurements

Information about sociodemographic and lifestyle aspects, education level, individual and family medical history, and current medication use was collected. Leisure-time PA was measured using the validated REGICOR questionnaire (23) which included questions to collect information about the type of activity, frequency (number of days) and duration (min/day) performed during a representative month.

Waist circumference was measured midway between the lowest rib and the iliac crest using an anthropometric tape, body weight was measured twice using high-quality electronic calibrated scales and height was measured twice using a wall-mounted stadiometer. Systolic and diastolic blood pressure was measured at rest 3 times using a validated semiautomatic oscillometer (Omron HEM-705CP, Kyoto, Japan) and the mean of repeated measures was used.

Blood samples were collected after an overnight fast. Tubes of serum and plasma were divided into aliquots, coded and stored at −80°C in a central laboratory until analyses. Plasma levels of glucose, insulin, total cholesterol, high density lipoprotein (HDL) cholesterol and triglycerides were measured using standard enzymatic methods, low density lipoprotein (LDL) cholesterol was calculated with the Friedewald formula (whenever triglycerides were <300 mg/dL).

### Stool samples collection, DNA extraction and 16S amplicon sequencing

Stool samples were collected at home by participants and kept frozen till the delivery to the laboratory. Stool samples were then separated into 250 mg aliquots and stored at −80°C, until analysis. Patients exposed to antibiotics treatment (*N* = 6) were excluded from the present analysis.

Microbial DNA was extracted using the QIAmp PowerFecal DNA kit (Qiagen, Hilden, Germany) following the manufacturer's instructions. Previous extraction samples were disrupted in a 5 min lysis step using FastPrep-24™ 5G Homogenizer (MP Biomedicals, Santa Ana, CA, USA). After extraction, the DNA was stored at −20°C until further processing. DNA concentration and purity were assessed with the Qubit 2.0 Fluorometer-dsDNA Broad Range (BR) Assay Kit (Thermo Fisher Scientific, Waltham, MA, USA).

The V4 region (515F-806R) of the prokaryotic 16S ribosomal RNA (rRNA) gene was amplified in triplicate PCR reactions. PCR was performed in a total reaction volume of 35 μl, and the master mix contained 0.7 μl of a unique barcoded primer, 515F-n and 806R-n (10 μM each per reaction), 0.7 μl dNTPs mixture, 0.35 μl Phusion Green Hot Start II High-Fidelity DNA Polymerase (2 U/μl; Thermo Scientific, Landsmeer, The Netherlands), 7 μl 5× Phusion Green HF Buffer, and 25.5 μl DNAse- RNAse-free water (Promega, Madison, WI, USA). The amplification program included 30 s of initial denaturation step at 98°C, followed by 25 cycles of denaturation at 98°C for 10 s, annealing at 50°C for 10 s, elongation at 72°C for 10 s, and a final extension step at 72°C for 7 min. The PCR products were visualized in 1% agarose gel (~290 bp) and purified with CleanPCR kit (CleanNA, Alphen Aan den Rijn, The Netherlands). The DNA concentration of the purified PCR products was measured with Qubit dsDNA BR Assay Kit, and 200 ng from each purified PCR product was used for the construction of the sequencing libraries, which were sequenced on an Illumina Novaseq platform. To ensure the quality of the sequencing data, artificial mock communities with known composition (24) were included in each library as positive controls, and 7 fecal samples were sequenced in duplicates. Finally, to control for potential contaminant sequences, negative control samples were included in each library, using as DNA template, nuclease free-water or material from DNA extraction blanks.

Raw sequence data were processed using the NG-Tax pipeline (24), with a read length of 100 nt. Paired-end libraries were demultiplexed and only read pairs with perfectly matching barcodes were used for downstream steps. Amplicon sequence variants (ASVs), were determined with the default settings and taxonomy was assigned to each ASV, using the USEARCH algorithm (25) and the Silva database (v138.1) (26).

### Statistical analysis

The clinical characteristics of the study participants were described using the software IBM SPSS Statistics version 23 (SPSS Inc., Chicago, Illinois, USA). Numerical variables were considered normally distributed according to the central limit theorem and described as means and standard deviations, whereas categorical variables were described as numbers and percentages. Differences across tertiles of UPF consumption (in % of kcal/day from total energy intake) were tested with Pearson's chi square test or one-way ANOVA as appropriate and *p* < 0.05 deemed as significant.

Fecal microbiota data was analyzed using R (version 4.0.5) and R Studio (version 1.2.5033). Prior filtering steps, a total of 192,011,642 sequence reads for 656 samples was obtained, clustering into 6285 ASVs. These ASVs represented 342 bacterial taxa at genus level. A cut off value of 10% of prevalence at genus level on the absolute abundances of the ASV counts was used to remove ASVs with a prevalence ≤10% between samples. After filtering steps, a total of 179,946,951 sequence reads for 645 samples and 91 genera represented were obtained. The minimum number of sequence reads detected in the dataset was 33, the maximum number of sequence reads was 988,905, the average number of sequence reads was 278,986.

The total relative abundance and percentage of each taxon per tertile was assessed at genus level. Principal component analysis (PCA) was calculated over centered log-ratio (CLR) transformed counts to evaluate the fecal microbiota distribution of the study population among the tertiles of UPF consumption. Fecal microbial richness and diversity was assessed by calculating Chao1, Shannon and Simpson indices on absolute abundance counts and differences across tertiles of UPF consumption tested with one-way ANOVA. Beta diversity was calculated in terms of Euclidean distance over CLR-transformed counts and permutational multivariate analysis of variance (PERMANOVA) performed to test differences in microbiota dissimilarity across tertiles of UPF consumption. The log-normalized Firmicutes-to-Bacteroidetes ratio was computed based on the relative abundance between the phylum Firmicutes and Bacteroidetes and differences across tertiles of UPF consumption tested using one-way ANOVA.

The association between tertiles of UPF consumption and fecal microbiota was assessed using the R package MaAsLin2 (27) (version 1.2.0). Specifically, counts were CLR-transformed, and model adjusted for different potential confounders (age, sex, recruiting center, smoking habits, diabetes prevalence, BMI categories (overweight, with BMI 27–29.9 kg/m^2^; obese, with BMI 30–39.9 kg/m^2^; severe obese, with BMI ≥40 kg/m^2^), and tertiles of PA. Multiple testing correction was performed using Benjamini-Hochberg procedure, and *p* < 0.2 deemed as significant. Spearman's correlation was used to test the relationship between taxa significantly associated with tertiles of UPF consumption, different G4 UPF items categories and cardiovascular risk factors: body weight, waist circumference, and body mass index (BMI), systolic blood pressure (SBP), diastolic blood pressure (DBP), fasting plasma glucose (FPG), insulin, total cholesterol, HDL cholesterol, LDL cholesterol, triglycerides, and glycated hemoglobin (HbA1c).

## Results

### General characteristics of the study population

A total of 656 participants in the framework of the PREDIMED-Plus clinical trial with available fecal microbiota data were included in this study. After applying quality filtering cutoffs and excluding those participants exposed to antibiotic treatment, the study population was reduced to 645 individuals stratified according to tertiles of UPF consumption. The characteristics of the study populations are described in Table 2. A total of 215 individuals were allocated in tertile 1 (T1) representing those individuals with lower consumption of UPF (7.2 ± 2.3%); 217 individuals in T2 (13.1 ± 1.6%) and 213 individuals T3 (21.4 ± 5.0%), representing those individuals with higher consumption of UPF. Participants in the highest tertiles of UPF (T2 and T3) consumption tended to be younger, with a higher energy consumption and lower adherence to MedDiet.

**Table 2:** Characteristics of the study population according to tertiles of UPF consumption.

**Tertiles of UPF consumption (% kcal/day from total energy intake)**	**T1 (*N =* 215)** **7.177 ±2.349**	**T2 (*N =* 217)** **13.094 ±1.572**	**T3 (*N =* 213)** **21.434 ±4.956**	***p*** **value^&^**
Women	103 (47.9)	99 (45.6)	102 (47.9)	0.861
Recruiting center				0.048
Alicante	51 (23.7)	49 (22.6)	45 (20.7)	
Barcelona IMIM	26 (12.1)	21 (9.7)	19 (8.9)	
Reus	119 (55.3)	124 (57.1)	110 (51.6)	
Valencia	19 (8.8)	23 (10.6)	41 (19.2)	
Smoking habits				0.659
Ex-smoker	81 (37.7)	87 (40.1)	84 (39.4)	
Never smoked	104 (48.4)	108 (49.8)	98 (46.0)	
Smoker	30 (14.0)	22 (10.1)	31 (14.6)	
Education				0.726
NA	1 (0.5)	1 (0.5)	0 (0.0)	
Higher education	40 (18.6)	45 (20.7)	32 (15.0)	
Primary school	113 (52.6)	109 (50.2)	119 (55.9)	
Secundary school	61 (28.4)	62 (28.6)	62 (29.1)	
Civil status				0.724
NA	0 (0.0)	0 (0.0)	1 (0.5)	
Divorced	14 (6.5)	10 (4.6)	17 (8.0)	
Married	161 (74.9)	174 (80.2)	160 (75.1)	
Separated	7 (3.3)	7 (3.2)	6 (2.8)	
Single	8 (3.7)	9 (4.1)	11 (5.2)	
Widowed	25 (11.6)	17 (7.8)	18 (8.5)	
BMI category				0.738
Overweight (27–29.9 kg/m^2^)	54 (25.1)	55 (25.3)	49 (23.0)	
Obese (30–39.9 kg/m^2^)	102 (47.4)	95 (43.8)	107 (50.2)	
Severe obese (≥ 40 kg/m^2^)	59 (27.4)	67 (30.9)	57 (26.8)	
Hypertension	204 (94.0)	206 (94.9)	198 (93.0)	0.692
Diabetes	60 (27.9)	48 (22.1)	53 (24.9)	0.381
Hypercholesterolemia	146 (67.9)	142 (65.4)	139 (65.3)	0.810
Age (years)	65.7 ± 4.9	64.8 ± 4.9^*^	64.5 ± 4.8^*^	0.023
Body weight (kg)	86.8 ± 13.1	87.2 ± 13.3	87.1 ± 12.9	0.943
Waist circumference (cm)	107.8 ± 10.5	107.7 ± 9.7	107.5 ± 9.9	0.962
BMI (kg/m^2^)	32.8 ± 3.5	32.9 ± 3.6	33.0 ± 3.5	0.906
FPG (mg/dL)	116.7 ± 22.2	116.9 ± 28.4	112.3 ± 21.6	0.087
Insulin (mU/mL)	19.8 ± 12.2	19.9 ± 11.7	19.2 ± 9.7	0.774
Total cholesterol (mg/dL)	200.4 ± 37.9	199.8 ± 37.9	201.4 ± 35.8	0.900
HDL cholesterol (mg/dL)	49.4 ± 10.4	49.9 ± 11.9	49.3 ± 12.1	0.853
LDL cholesterol (mg/dL)	122.2 ± 34.1	117.6 ± 30.8	121.8 ± 31.0	0.268
Triglycerides (mg/dL)	152.8 ± 74.0	164.0 ± 93.2	167.4 ± 111.9	0.245
Glycated hemoglobin (%)	6.1 ± 0.8	6.1 ± 0.9	6.0 ± 0.8	0.668
UPF energy intake (kcal/day)	171.6 ± 70.6	329.6 ± 77.3^*^	578.0 ± 234.3^*^^†^	< 0.001
Total energy intake (kcal/day)	2363.4 ± 482.1	2516.1 ± 499.1^*^	2677.2 ± 482.1^*^^†^	< 0.001
Adherence to MedDiet score	8.7 ± 2.4	8.1 ± 2.6^*^	7.7 ± 2.3^*^	< 0.001

UPF, ultra-processed food; NA, not available, BMI, body mass index; FPG, fasting plasma glucose; HDL, high density lipoprotein; HDL, low density lipoprotein. Data expressed in number (percentage) for categorical variables and mean ± standard deviation for numerical variables.

& Differences across tertiles calculated with Pearson's chi square or one-way ANOVA, differences between tertiles calculated with Pearson's chi square or Student's t-test.

* p < 0.05 vs. T1;

† p < 0.05 vs. T2.

**Figure 1:**
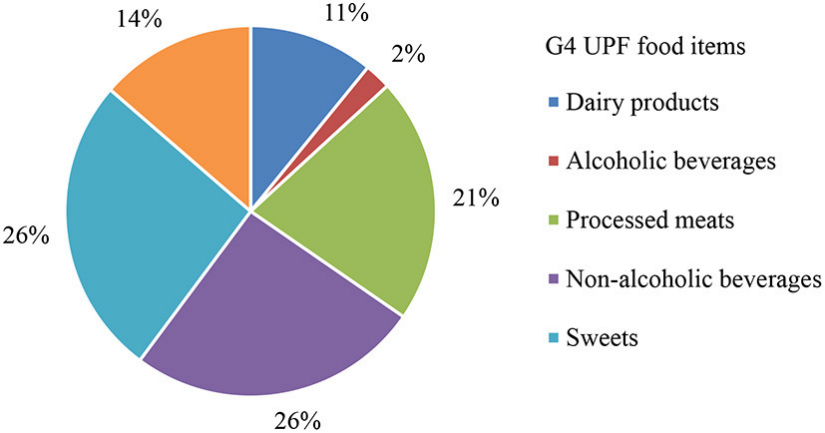
Pie chart showing the proportion (in % kcal/day) of UPF consumption (group 4 of the NOVA classification system) in the total diet of the study population.

From the total energy of UPF consumed, the main contributors (Figure 1) were represented by non-alcoholic beverages (26%), sweets (26%), processed meats (21%), preprepared dishes, snacks and fast-foods (14%), dairy products (11%) and alcoholic beverages (2%).

### Fecal microbiota diversity and dissimilarity and main phyla distribution across study groups

No statistically significant differences in alpha diversity indices calculated, Chao1, Shannon, and Simpson, across tertiles of UPF consumption (Supplementary Figure 1 and Supplementary Table 1) were observed. PCA over the CLR-transformed taxonomic feature counts shown that PC1 and PC2 account ~10.15% and 8% to the total variation, respectively. PCA plot showed that the fecal microbiota samples did not cluster based on tertiles of UPF consumption (Supplementary Figure 2). Furthermore, the result of the PERMANOVA test based on Euclidean distance did not show statistically significant differences across tertiles of percentage in UPF energy intake (Supplementary Table 2). There were no differences in Firmicutes-to-Bacteroidetes ratio across tertiles UPF consumption (Supplementary Table 3).

### Association between specific fecal microbiota taxa and UPF consumption

The analysis performed with MaAsLin2 shown a positive association between fecal microbiota genera *Alloprevotella* and *Sutterella*, representing, respectively, 1.2% and 0.6% of the total relative abundance (Supplementary Table 4), and T2 of percentage in UPF energy intake, and a positive association between fecal microbiota genera *Alloprevotella, Negativibacillus*, and *Prevotella*, representing respectively 0.3%, 0.03% and 0.3% of the total relative abundance (Supplementary Table 4), and T3 of percentage in UPF energy intake (Table 3).

**Table 3:** Results of the association analysis performed with MaAsLin2 between taxonomic feature counts at genus level and tertiles of percentage in UPF energy intake.

**Feature**	**Tertile^#^**	**coef**	**stderr**	**pval**	**padj**
*Alloprevotella*	2	0.430	0.133	0.001	**0.041**
	3	0.410	0.134	0.002	**0.065**
*Negativibacillus*	2	0.071	0.058	0.224	0.725
	3	0.170	0.059	0.004	**0.096**
*Prevotella*	2	0.161	0.119	0.178	0.687
	3	0.335	0.121	0.006	**0.116**
*Sutterella*	2	0.330	0.119	0.006	**0.116**
	3	0.093	0.120	0.441	0.841

Association tested with GLM adjusted for age, sex, recruiting center, smoking habits, diabetes prevalence, BMI categories, tertiles of physical activity. Multiple testing correction was performed using Benjamini-Hochberg procedure and p < 0.2 deemed as significant.

# (reference: Tertile 1). Significant associations are indicated in bold.

In addition, the results of the Spearman correlation did not show any relevant association between taxa significantly associated with UPF consumption and UPF items categories (Supplementary Figure 3 and Supplementary Table 5) nor with cardiovascular risk factors (Supplementary Figure 4 and Supplementary Table 6).

## Discussion

The current cross-sectional study aimed to assess the association between UPF consumption and fecal microbiota composition in senior subjects with overweight/obesity and MetS within the frame of the PREDIMED-Plus trial. A previous study conducted in the whole PREDIMED-Plus cohort shown that higher consumption of UPF was associated with higher visceral and overall adiposity accumulation (28). Similar results have also been reported some years before by Mendoça et al., in the context of a prospective cohort study in healthy middle-aged Spanish individuals, in which those participants in the highest quartile of UPF consumption showed an increased risk of developing overweight or obesity (29). A recent comprehensive systematic review explored extant relevant nutrition literature reporting increasing evidence of UPF exposure associated with adverse health outcomes such as overweight, obesity and cardio-metabolic risk (30), suggesting that a high frequency of UPF consumption could be related to obesity and several cardiometabolic alterations.

In the present study, the population was categorized according to tertiles of UPF consumption, and we observed that subjects allocated in the highest tertile presented lower adherence to MedDiet and higher total energy intake. These results were in line with the aforementioned articles, and very similar to the ones obtained previously by Cuevas-Sierra et al. where higher values of total energy intake and a lower adherence to MedDiet were observed in subjects consuming more than 5 UPF servings/day (18).

According to our findings, we did not observe any association between alpha and beta diversity nor with Firmicutes-to-Bacteroidetes ratio in our study population. In relation to that, it has been reported that food processing may disturb gut microbiota composition also reducing bacterial diversity. However, the mechanisms by which UPF affects gut microbial homeostasis remain unclear (31). In addition, there are studies linking a varied and omnivorous diet, and consequently higher adherence to MedDiet, with an increase in the ratio Firmicutes-to-Bacteroidetes (32, 33). In line with these studies, Partula et al. aimed to explore the association between usual diet and gut microbiota composition, established through 16S rRNA gene sequencing in stool samples from healthy French adults (34). They observed that food items for which a limited consumption is generally recommended (fried products, sodas or sugary drinks, fatty sweet products, processed meats, ready-cooked meals, desserts, and cheese) were negatively associated with alpha-diversity and contributed to shifts within microbiota composition (beta-diversity). These contradictory results in relation to the aforementioned studies can be explained by a lower consumption of UPF in our senior study population.

In the present study we observed a positive association between 4 different taxa abundances and higher UPF consumption. Specifically, we found that abundance of genera *Alloprevotella, Negativibacillus, Prevotella* and *Sutterella*, was positively associated with the highest consumption of UPF. These positive associations are in accordance with other recently published studies. The association between specific food items and relative abundances of taxa tested by Partula et al. shown that *Alloprevotella* was negatively associated with the consumption of fruits (34). *Alloprevotella* was found enriched in the gut microbial community determined by absolute quantification 16S rRNA gene amplicon sequencing of healthy individuals exposed to 4 days dietary fiber intervention (35). However, these findings should be interpreted with caution as obtained in a small sample size.

*Negativibacillus* has been recently discovered in the microbial community of human stool samples and it has been found enriched in patients with ulcerative colitis (UC) (36). There is not much information on this genus in the bibliography, however, in a previous published study it has been found that the abundance of *Negativibacillus* was positively associated with obesity in mice (37).

*Prevotella* species, as *Negativibacillus*, have been associated with patients with UC (36). This genus has also been associated with plant-based diet, rich in fiber and carbohydrates, and has been considered as a biomarker of health. Nevertheless, there are important confounding factors such as microbial exposure, lifestyle and geography that may affect this associative evidence (38). In addition, the potential pathogenic role of certain *Prevotella* strains has been also highlighted (39). The strain-level diversity may explain the controversial role of *Prevotella* species in health and disease (40). This could partially explain the association that we found between this taxon and higher UPF consumption, as the response to different dietary patterns may be strain dependent and undetectable due to the next generation sequencing techniques utilized that limited us to genus level resolution, as also emphasized by De Filippis et al. (40).

Finally, *Sutterella*, has been recently linked to inflammatory gastrointestinal diseases (41–44). The relationship between diet and development of inflammatory gastrointestinal tract disorders has been indicated in several epidemiological studies (45). Previous meta-analysis studies showed that soft drink consumption and sucrose intake were associated with 69% and 10% increased risk of UC development, respectively (46, 47). In addition, consumption of fruits and vegetables was related to decreased UC development (48). This could in part explain the positive association between *Sutterella* and UPF consumption in our cohort.

Despite these findings, in the current study we did not observe any correlation between the above-mentioned UPF-associated taxa, UPF items categories and CVD risk factors, respectively. Nevertheless, lower adherence to MedDiet was observed in those participants in the highest tertile of UPF energy consumption. These results were in consonance with previous studies (49–51).

Our study has some limitations that deserve to be discussed. First, the observational design of the study did not make possible to conclude on causality or directionality as these results indicate just associations. However, observational design is the most indicated approach to explore these type of associations, as longitudinal studies based on interventions including UPF intake may be detrimental for health and could cause ethical issues. Secondly, the results cannot be extrapolated to other populations since participants included in the analysis were senior Mediterranean subjects with overweight/obesity and MetS. Under the methodological point of view, the nature of 16S sequencing limits the taxonomic profiling to genus-level resolution. Finally, the assessment of food intake through a FFQ is sensitive to possible measurements errors. Nevertheless, food-based FFQs have been widely used as a tool in epidemiological studies since the 1990's (52).

On the other hand, the current study also has some strengths, such as the large cohort sample size, the extensive data collection, and the use of several potential confounding factors which minimizes the possibility of reverse causality bias. Finally, it is also important to highlight that the study setup provides an ideal scenario to explore specific gut microbial profiles related to dietary patterns.

## Conclusions

In conclusion, the results of this present observational study conducted within the frame of the PREDIMED-Plus cohort revealed that the consumption of UPF (rich in energy density, added sugars, salt, and saturated and trans fatty acids, and low in fiber, protein, and micronutrients) was positively associated with lower adherence to MedDiet and higher total energy intake. In addition, we observed a positive association with specific taxa (*Alloprevotella, Negativibacillus, Prevotella* and *Sutterella*) related to inflammatory gastro-intestinal diseases and low consumption of fruits and vegetables, among others, across tertiles of UPF consumption.

Diet and nutritional status are important determinants in human health. The role of diet in shaping the gut microbiota could be key to improve host's health. Consequently, further future studies including whole metagenome shotgun sequencing would be crucial to obtain more detailed taxonomic identification. Furthermore, information generated from metabolomics analysis would be useful to improve and strengthen the results. In addition, more in-depth studies regarding the association between taxa and UPF consumption are needed in order to further reduce the controversies between the authors. The detection of unhealthy dietary patterns and association with gut microbial profiles would be essential for preventing related chronic diseases and to improve public health strategies.

## Data availability statement

The datasets generated and analyzed during the current study are not publicly available due to data regulations and for ethical reasons, considering that this information might compromise research participants' consent because our participants only gave their consent for the use of their data by the original team of investigators. However, collaboration for data analyses can be requested by sending a letter to the PREDIMED-Plus steering Committee (predimed_plus_scommittee@googlegroups.com). The request will then be passed to all the members of the PREDIMED-Plus Steering Committee for deliberation.

## Ethics statement

The studies involving human participants were reviewed and approved by the study was conducted according to the guidelines of the Declaration of Helsinki, and approved by the Hospital Universitari Sant Joan de Reus Ethics Committee (protocol code 13-07-25/7proj2, date of approval: 25/07/2013), and the Comité de Ética de la Investigación Provincialde Málaga (protocol code Predimed+DM/01, date of approval: 27/11/ 2014). The patients/participants provided their written informed consent to participate in this study. The patients/participants provided their written informed consent to participate in this study.

## Author contributions

All the principal PREDIMED-Plus investigators from the respective centers contributed to study concept and design and to data extraction from the participants (FT, JVio, DC, MF, JVid, and JS-S). AA and MM performed the statistical analyses. AA, MM, and JS-S drafted the manuscript. All authors reviewed the manuscript for important intellectual content and approved the final version to be published.

## Funding

This work was supported by the official Spanish Institutions for funding scientific biomedical research, CIBER Fisiopatología de la Obesidad y Nutrición (CIBEROBN) and Instituto de Salud Carlos III (ISCIII), through the Fondo de Investigación para la Salud (FIS), which is co-funded by the European Regional Development Fund (three coordinated FIS projects leaded by JS-S, including the following projects: PI13/00462, PI16/00501 and PI19/00576); The Generalitat Valenciana PROMETEO 17/2017, PROMETEO 21/2021 and APOSTD/2020/164 (to AÁ-S). This research was also partially funded by EU-H2020 Grant Eat2beNICE/H2020-SFS-2016-2 and by NIH grant R01DK127601. None of the funding sources took part in the design, collection, analysis, interpretation of the data, or writing the report, or in the decision to submit the manuscript for publication. Food companies Hojiblanca (Lucena, Spain) and Patrimonio Comunal Olivarero (Madrid, Spain) donated extra virgin olive oil; and the Almond Board of California (Modesto, CA), American Pistachio Growers (Fresno, CA), and Paramount Farms (Wonderful Company, LLC, Los Angeles, CA) donated nuts for the PREDIMED-Pilot study. AA was supported by the European Union's Horizon 2020 research and innovation programme under the Marie Skłodowska-Curie grant agreement No. 713679 and from the Universitat Rovira i Virgili (URV). MM was supported by Sara Borrell -CD21/00045- Instituto de Salud Carlos III (ISCIII). IM-I was supported by a Miguel Servet II contract -CPII21-00013- Instituto de Salud Carlos III (ISCIII).

## Conflict of interest

The authors declare that the research was conducted in the absence of any commercial or financial relationships that could be construed as a potential conflict of interest.

## Publisher's note

All claims expressed in this article are solely those of the authors and do not necessarily represent those of their affiliated organizations, or those of the publisher, the editors and the reviewers. Any product that may be evaluated in this article, or claim that may be made by its manufacturer, is not guaranteed or endorsed by the publisher.
