# Supplementary material for: Association between ultra-processed food consumption and gut microbiota in senior subjects with overweight/obesity and metabolic syndrome
Source: Front Nutr. 2022 Oct 10;9:976547. doi: 10.3389/fnut.2022.976547 (PMC9589409; doi:10.3389/fnut.2022.976547)
Supplement: Supplementary file 1 [file Data_Sheet_1.docx]

Supplementary Material

**Supplementary Figure 1**. Box plot showing differences in Chao1, Shannon and Simpson indices between tertiles of UPF consumption.

**
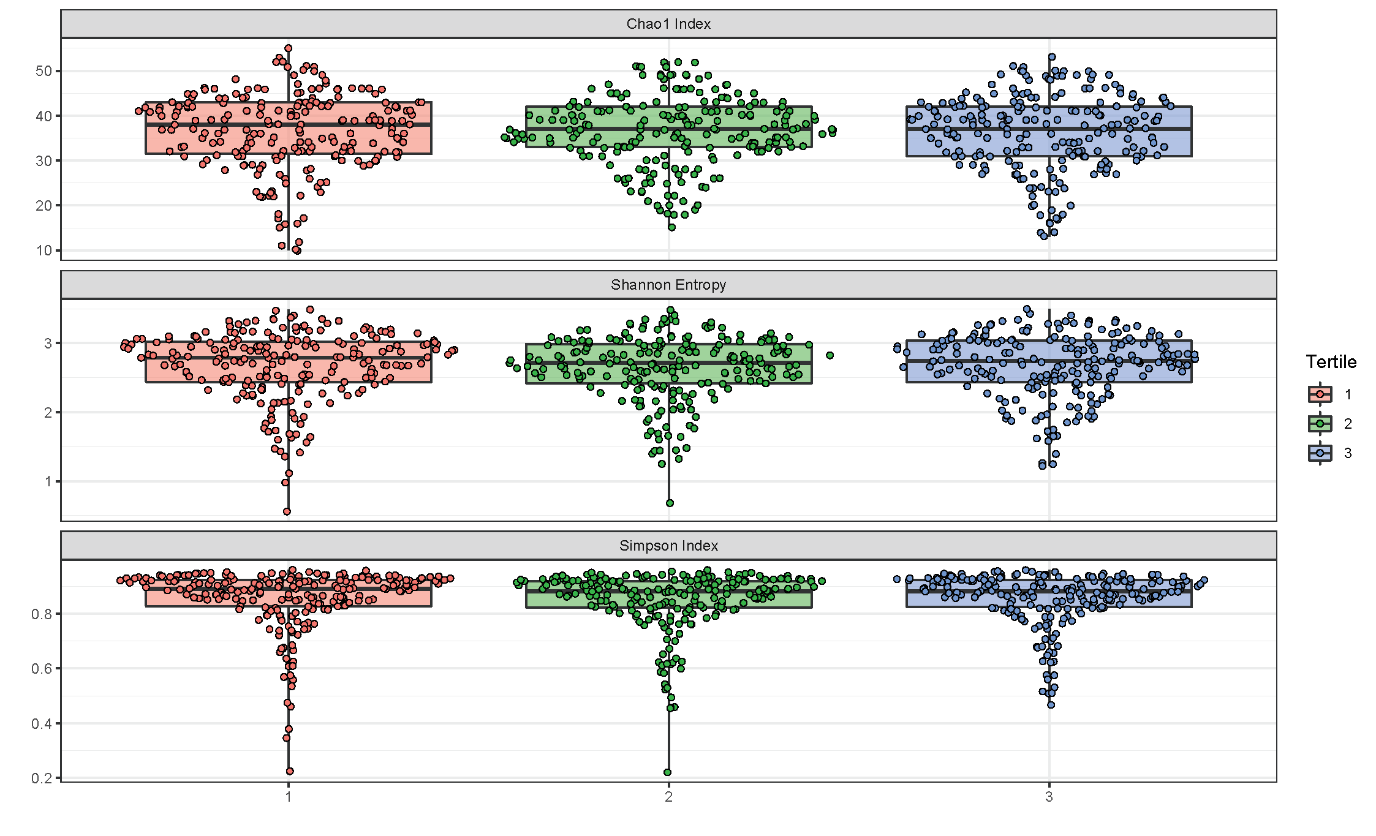
**

**Supplementary Table 1**. Differences in alpha diversity indices Chao1, Simpson, Shannon, between tertiles of UPF consumption.

| **Index** | **Tertile 1** | **Tertile 2** | **Tertile 3** | ***p* value** |
| --- | --- | --- | --- | --- |
| Chao1 | 36.5 ± 8.6 | 36.4 ± 7.9 | 36.7 ± 8.5 | 0.986 |
| Simpson | 0.9 ± 0.1 | 0.8 ± 0.1 | 0.9 ± 0.1 | 0.678 |
| Shannon | 2.7 ± 0.5 | 2.6 ± 0.5 | 2.7 ± 0.5 | 0.787 |

Data presented as mean ± SD. One-way ANOVA used to test differences across tertiles of UPF consumption (in % from total energy intake/day); p < 0.05 deemed as significant.

**Supplementary Figure 2**. Principal component analysis plot of the fecal microbiota distribution according to tertiles of UPF consumption.


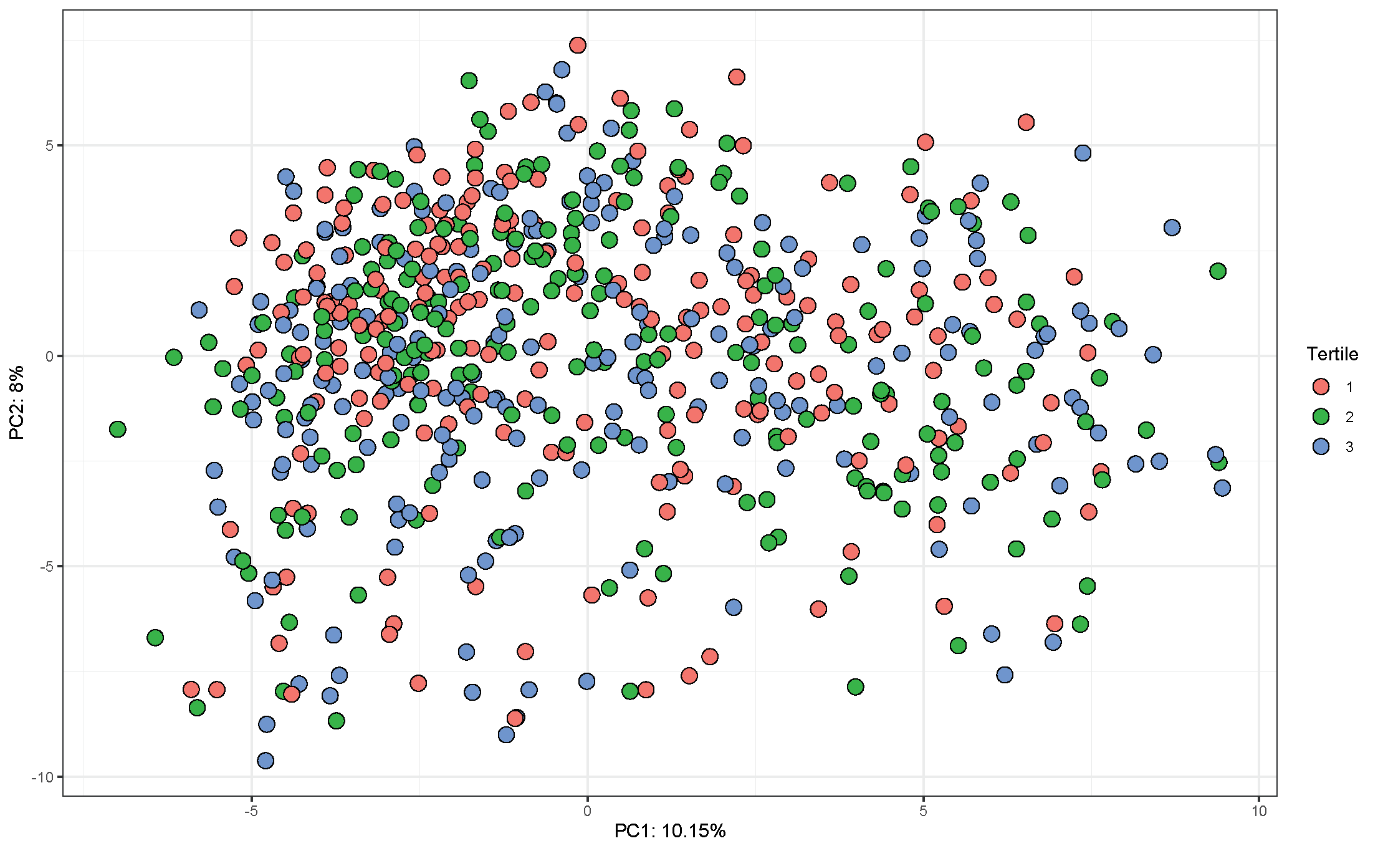


**Supplementary Table 2**. Results of PERMANOVA test based on Euclidean distance.

|  | **Df** | **Sums of squares** | **Mean squares** | **F.Model** | **R2** | **Pr(>F)** |
| --- | --- | --- | --- | --- | --- | --- |
| Tertiles | 2 | 307 | 153.64 | 1.2168 | 0.00364 | 0.122877 |
| recruiting_center | 3 | 2693 | 897.50 | 7.1081 | 0.03187 | 0.000999 |
| sex | 1 | 288 | 288.23 | 2.2827 | 0.00341 | 0.001998 |
| age | 1 | 221 | 220.94 | 1.7498 | 0.00262 | 0.015984 |
| smoking_habits | 2 | 344 | 172.10 | 1.3630 | 0.00407 | 0.046953 |
| diabetes | 1 | 526 | 525.89 | 4.1650 | 0.00623 | 0.000999 |
| BMI_categories | 2 | 335 | 167.72 | 1.3283 | 0.00397 | 0.049950 |
| PA_categories | 2 | 214 | 106.76 | 0.8455 | 0.00253 | 0.812188 |
| Residuals | 630 | 79547 | 126.26 |  | 0.94166 |  |
| Total | 644 | 84475 |  |  | 1.00000 |  |

**Supplementary Table 3**. Differences in Firmicutes-to-Bacteroidetes ratio across tertiles of UPF consumption.

| **Index** | **Tertile 1** | **Tertile 2** | **Tertile 3** | ***p* value** |
| --- | --- | --- | --- | --- |
| FB ratio | 0.7 ± 1.6 | 0.5 ± 1.4 | 0.8 ± 1.9 | 0.103 |

Data presented as mean ± SD. One-way ANOVA used to test differences across tertiles UPF (in % from total energy intake/day); p < 0.05 deemed as significant.

**Supplementary Figure 3**. Heatmap describing the correlation between taxa significantly associated with UPF consumption and specific UPF items categories.


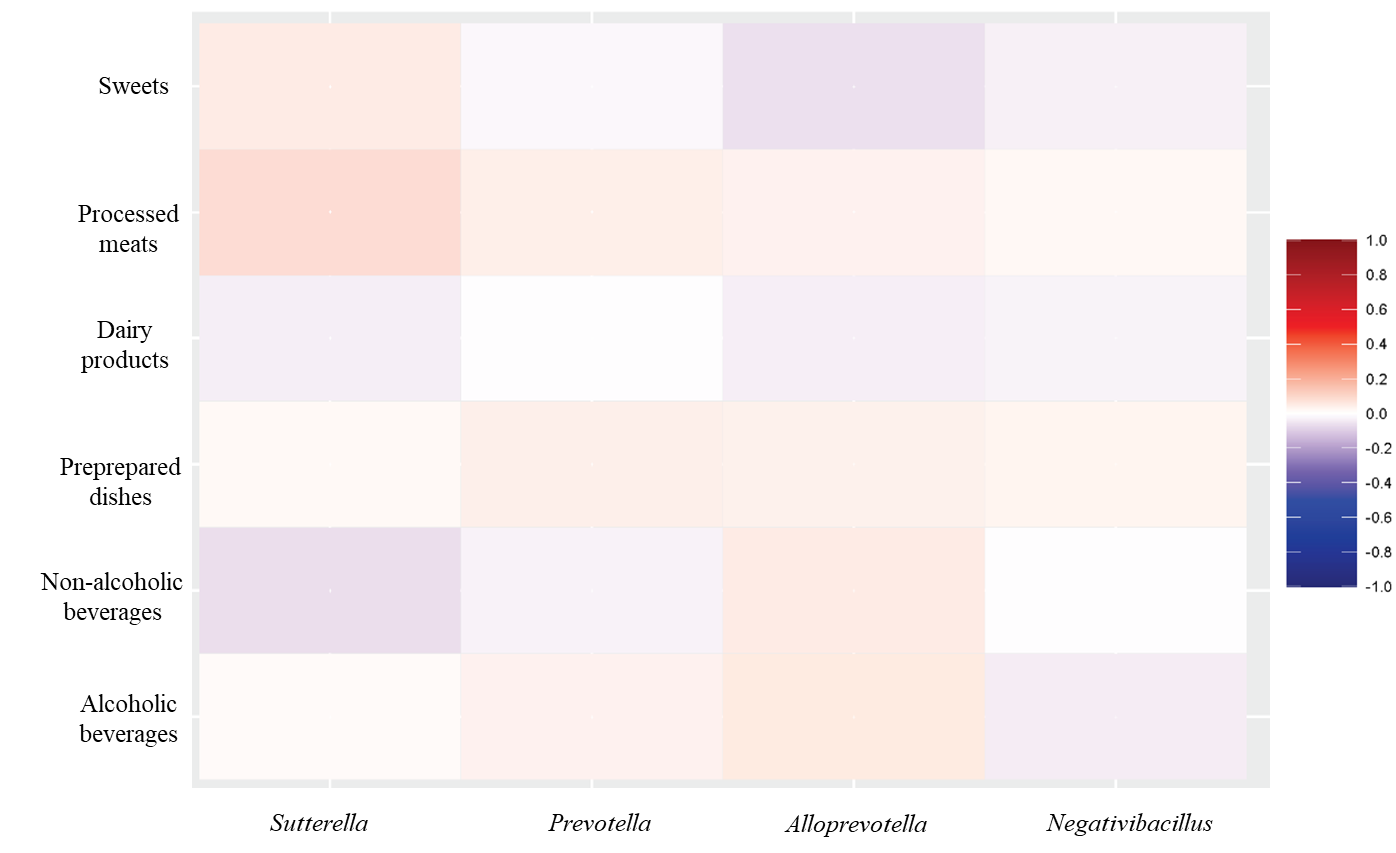


**Supplementary Table 4.** Total relative abundance and percentage of each genus per tertile of UPF consumption

| **Genus** | **T1 abundance** | **T1 frequency** | **T2 abundance** | **T2 frequency** | **T3 abundance** | **T3 frequency** |
| --- | --- | --- | --- | --- | --- | --- |
| Acidaminococcus | 0.0031 | 0.31% | 0.0031 | 0.31% | 0.0023 | 0.23% |
| Adlercreutzia | 0.0003 | 0.03% | 0.0004 | 0.04% | 0.0009 | 0.09% |
| Agathobacter | 0.0007 | 0.07% | 0.0009 | 0.09% | 0.0007 | 0.07% |
| Akkermansia | 0.0206 | 2.06% | 0.0248 | 2.48% | 0.0192 | 1.92% |
| Alistipes | 0.0244 | 2.44% | 0.0210 | 2.10% | 0.0190 | 1.90% |
| Alloprevotella | 0.0030 | 0.30% | 0.0122 | 1.22% | 0.0099 | 0.99% |
| Anaerostipes | 0.0175 | 1.75% | 0.0165 | 1.65% | 0.0230 | 2.30% |
| Bacteroides | 0.1893 | 18.93% | 0.1838 | 18.38% | 0.1529 | 15.29% |
| Barnesiella | 0.0114 | 1.14% | 0.0085 | 0.85% | 0.0089 | 0.89% |
| Bifidobacterium | 0.0249 | 2.49% | 0.0237 | 2.37% | 0.0355 | 3.55% |
| Bilophila | 0.0011 | 0.11% | 0.0015 | 0.15% | 0.0015 | 0.15% |
| Blautia | 0.0605 | 6.05% | 0.0554 | 5.54% | 0.0741 | 7.41% |
| Butyricicoccus | 0.0016 | 0.16% | 0.0014 | 0.14% | 0.0014 | 0.14% |
| Butyricimonas | 0.0014 | 0.14% | 0.0011 | 0.11% | 0.0012 | 0.12% |
| Butyrivibrio | 0.0027 | 0.27% | 0.0024 | 0.24% | 0.0036 | 0.36% |
| Catenibacterium | 0.0019 | 0.19% | 0.0126 | 1.26% | 0.0112 | 1.12% |
| Christensenellaceae_R-7_group | 0.0107 | 1.07% | 0.0160 | 1.60% | 0.0160 | 1.60% |
| Clostridium_sensu_stricto_1 | 0.0183 | 1.83% | 0.0049 | 0.49% | 0.0050 | 0.50% |
| Collinsella | 0.0053 | 0.53% | 0.0035 | 0.35% | 0.0056 | 0.56% |
| Coprococcus | 0.0031 | 0.31% | 0.0099 | 0.99% | 0.0101 | 1.01% |
| Desulfovibrio | 0.0109 | 1.09% | 0.0036 | 0.36% | 0.0026 | 0.26% |
| Dialister | 0.0042 | 0.42% | 0.0121 | 1.21% | 0.0098 | 0.98% |
| Dorea | 0.0103 | 1.03% | 0.0120 | 1.20% | 0.0145 | 1.45% |
| Erysipelotrichaceae_UCG-003 | 0.0117 | 1.17% | 0.0123 | 1.23% | 0.0133 | 1.33% |
| Escherichia-Shigella | 0.0147 | 1.47% | 0.0192 | 1.92% | 0.0106 | 1.06% |
| Eubacterium_eligens_group | 0.0215 | 2.15% | 0.0083 | 0.83% | 0.0077 | 0.77% |
| Eubacterium_hallii_group | 0.0116 | 1.16% | 0.0137 | 1.37% | 0.0207 | 2.07% |
| Eubacterium_ruminantium_group | 0.0177 | 1.77% | 0.0030 | 0.30% | 0.0032 | 0.32% |
| Eubacterium_siraeum_group | 0.0031 | 0.31% | 0.0032 | 0.32% | 0.0031 | 0.31% |
| Eubacterium_ventriosum_group | 0.0033 | 0.33% | 0.0008 | 0.08% | 0.0015 | 0.15% |
| Eubacterium_xylanophilum_group | 0.0010 | 0.10% | 0.0009 | 0.09% | 0.0008 | 0.08% |
| Faecalibacterium | 0.0015 | 0.15% | 0.0590 | 5.90% | 0.0543 | 5.43% |
| Family_XIII_AD3011_group | 0.0640 | 6.40% | 0.0005 | 0.05% | 0.0006 | 0.06% |
| Fusicatenibacter | 0.0005 | 0.05% | 0.0097 | 0.97% | 0.0092 | 0.92% |
| Haemophilus | 0.0101 | 1.01% | 0.0031 | 0.31% | 0.0019 | 0.19% |
| Holdemanella | 0.0040 | 0.40% | 0.0064 | 0.64% | 0.0096 | 0.96% |
| Incertae_Sedis | 0.0065 | 0.65% | 0.0003 | 0.03% | 0.0007 | 0.07% |
| Intestinibacter | 0.0005 | 0.05% | 0.0039 | 0.39% | 0.0032 | 0.32% |
| Lachnoclostridium | 0.0035 | 0.35% | 0.0039 | 0.39% | 0.0040 | 0.40% |
| Lachnospira | 0.0030 | 0.30% | 0.0051 | 0.51% | 0.0045 | 0.45% |
| Lachnospiraceae__CAG-56 | 0.0061 | 0.61% | 0.0018 | 0.18% | 0.0017 | 0.17% |
| Lachnospiraceae_ND3007_group | 0.0022 | 0.22% | 0.0019 | 0.19% | 0.0018 | 0.18% |
| Lachnospiraceae_NK4A136_group | 0.0113 | 1.13% | 0.0101 | 1.01% | 0.0088 | 0.88% |
| Lachnospiraceae_UCG-001 | 0.0018 | 0.18% | 0.0013 | 0.13% | 0.0015 | 0.15% |
| Megamonas | 0.0067 | 0.67% | 0.0084 | 0.84% | 0.0071 | 0.71% |
| Methanobrevibacter | 0.0038 | 0.38% | 0.0019 | 0.19% | 0.0033 | 0.33% |
| Mogibacterium | 0.0009 | 0.09% | 0.0006 | 0.06% | 0.0008 | 0.08% |
| Monoglobus | 0.0015 | 0.15% | 0.0013 | 0.13% | 0.0015 | 0.15% |
| Negativibacillus | 0.0003 | 0.03% | 0.0006 | 0.06% | 0.0010 | 0.10% |
| Odoribacter | 0.0042 | 0.42% | 0.0007 | 0.07% | 0.0010 | 0.10% |
| Oscillospiraceae__NK4A214_group | 0.0010 | 0.10% | 0.0039 | 0.39% | 0.0041 | 0.41% |
| Oscillospiraceae__UCG-002 | 0.0146 | 1.46% | 0.0267 | 2.67% | 0.0254 | 2.54% |
| Oscillospiraceae__UCG-003 | 0.0114 | 1.14% | 0.0010 | 0.10% | 0.0010 | 0.10% |
| Oscillospiraceae__UCG-005 | 0.0054 | 0.54% | 0.0049 | 0.49% | 0.0041 | 0.41% |
| Parabacteroides | 0.0154 | 1.54% | 0.0164 | 1.64% | 0.0177 | 1.77% |
| Paraprevotella | 0.0033 | 0.33% | 0.0103 | 1.03% | 0.0104 | 1.04% |
| Parasutterella | 0.0089 | 0.89% | 0.0030 | 0.30% | 0.0045 | 0.45% |
| Phascolarctobacterium | 0.0794 | 7.94% | 0.0133 | 1.33% | 0.0141 | 1.41% |
| Prevotella | 0.0145 | 1.45% | 0.0063 | 0.63% | 0.0091 | 0.91% |
| Prevotella_7 | 0.0034 | 0.34% | 0.0023 | 0.23% | 0.0048 | 0.48% |
| Prevotella_9 | 0.0035 | 0.35% | 0.1127 | 11.27% | 0.1008 | 10.08% |
| Prevotellaceae_NK3B31_group | 0.0165 | 1.65% | 0.0157 | 1.57% | 0.0209 | 2.09% |
| Rikenellaceae_RC9_gut_group | 0.0306 | 3.06% | 0.0029 | 0.29% | 0.0033 | 0.33% |
| Romboutsia | 0.0028 | 0.28% | 0.0041 | 0.41% | 0.0052 | 0.52% |
| Roseburia | 0.0074 | 0.74% | 0.0170 | 1.70% | 0.0162 | 1.62% |
| Ruminococcus | 0.0012 | 0.12% | 0.0303 | 3.03% | 0.0333 | 3.33% |
| Ruminococcus_gauvreauii_group | 0.0110 | 1.10% | 0.0027 | 0.27% | 0.0033 | 0.33% |
| Ruminococcus_torques_group | 0.0230 | 2.30% | 0.0070 | 0.70% | 0.0090 | 0.90% |
| Senegalimassilia | 0.0043 | 0.43% | 0.0007 | 0.07% | 0.0014 | 0.14% |
| Streptococcus | 0.0011 | 0.11% | 0.0109 | 1.09% | 0.0139 | 1.39% |
| Subdoligranulum | 0.0009 | 0.09% | 0.0197 | 1.97% | 0.0207 | 2.07% |
| Sutterella | 0.0006 | 0.06% | 0.0064 | 0.64% | 0.0046 | 0.46% |
| Terrisporobacter | 0.0262 | 2.62% | 0.0011 | 0.11% | 0.0014 | 0.14% |
| Turicibacter | 0.0008 | 0.08% | 0.0007 | 0.07% | 0.0016 | 0.16% |
| Tyzzerella | 0.0050 | 0.50% | 0.0007 | 0.07% | 0.0008 | 0.08% |
| uncultured_bacterium | 0.0324 | 6.19% | 0.0385 | 6.35% | 0.0318 | 6.03% |
| Veillonella | 0.0017 | 0.17% | 0.0028 | 0.28% | 0.0013 | 0.13% |

**Supplementary Figure 4**. Heatmap describing the correlation between taxa significantly associated with UPF consumption and specific CVD variables.


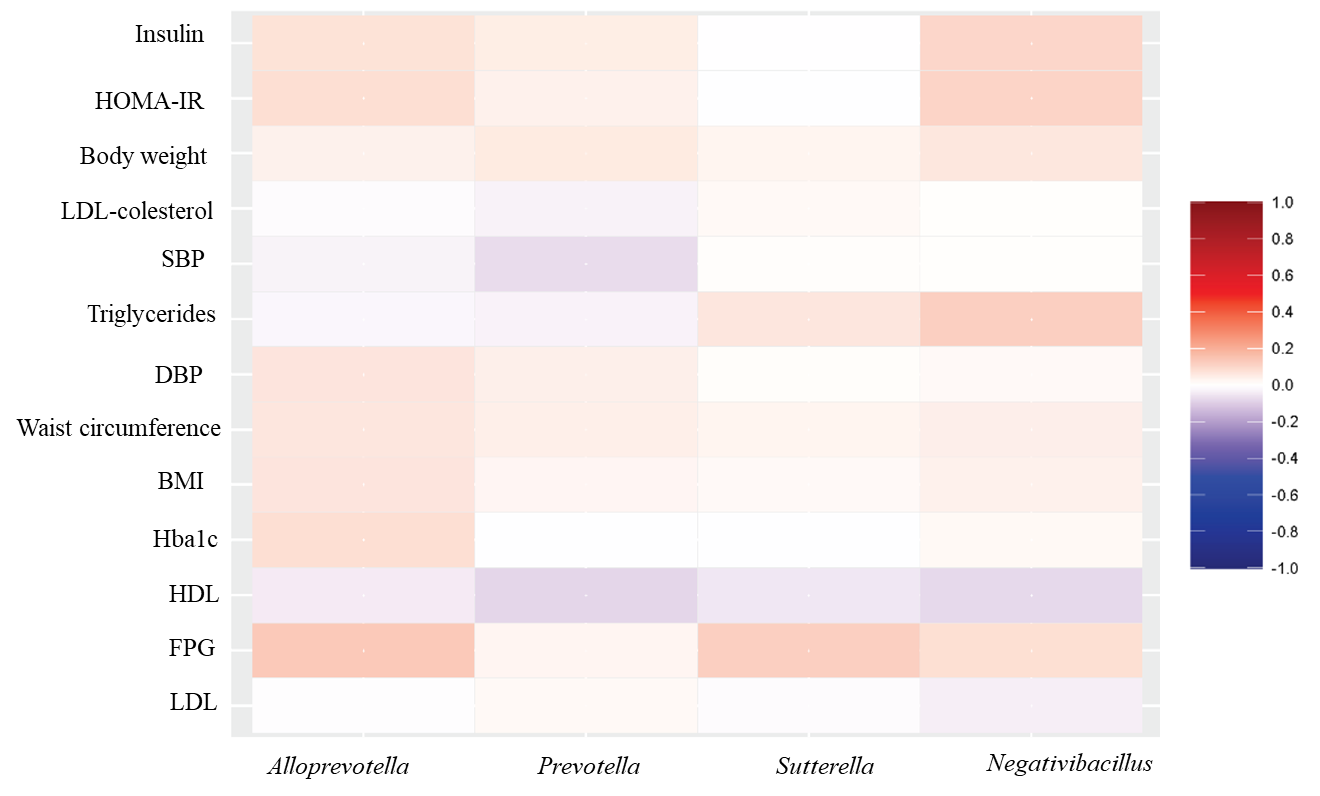


**Supplementary Table 5**. Results of the Spearman’s correlation between taxa significantly associated with UPF consumption and specific UPF items categories.

| **Taxa** | **UPF items groups** | **Correlation** | **p.adj** |
| --- | --- | --- | --- |
| Sutterella | processed_meats | 0,086 | 0,542 |
| Alloprevotella | alcoholic_beverages | 0,058 | 0,542 |
| Sutterella | sweets | 0,053 | 0,542 |
| Alloprevotella | non_alcoholic_beveragesic_beverages | 0,051 | 0,542 |
| Prevotella | processed_meats | 0,048 | 0,542 |
| Prevotella | preprepared_dishes | 0,045 | 0,542 |
| Alloprevotella | preprepared_dishes | 0,043 | 0,542 |
| Prevotella | alcoholic_beverages | 0,037 | 0,542 |
| Alloprevotella | processed_meats | 0,037 | 0,542 |
| Negativibacillus | preprepared_dishes | 0,035 | 0,542 |
| Negativibacillus | processed_meats | 0,026 | 0,649 |
| Sutterella | preprepared_dishes | 0,021 | 0,691 |
| Sutterella | alcoholic_beverages | 0,015 | 0,772 |
| Prevotella | dairy | -0,008 | 0,840 |
| Negativibacillus | non_alcoholic_beveragesic_beverages | -0,009 | 0,840 |
| Prevotella | sweets | -0,020 | 0,691 |
| Negativibacillus | dairy | -0,033 | 0,542 |
| Prevotella | non_alcoholic_beveragesic_beverages | -0,033 | 0,542 |
| Negativibacillus | sweets | -0,035 | 0,542 |
| Alloprevotella | dairy | -0,038 | 0,542 |
| Sutterella | dairy | -0,039 | 0,542 |
| Negativibacillus | alcoholic_beverages | -0,043 | 0,542 |
| Alloprevotella | sweets | -0,069 | 0,542 |
| Sutterella | non_alcoholic_beveragesic_beverages | -0,070 | 0,542 |

**Supplementary Table 6**. Results of the Spearman’s correlation between taxa significantly associated with UPF consumption and CVD variables.

| **Taxa** | **CVD variable** | **Correlation** | **p.adj** |
| --- | --- | --- | --- |
| Alloprevotella | FPG | 0,139 | 0,027 |
| Sutterella | FPG | 0,123 | 0,041 |
| Negativibacillus | triglycerides | 0,120 | 0,041 |
| Negativibacillus | HOMA-IR | 0,111 | 0,097 |
| Negativibacillus | insulin | 0,101 | 0,118 |
| Alloprevotella | HOMA-IR | 0,085 | 0,215 |
| Alloprevotella | hba1c | 0,084 | 0,215 |
| Negativibacillus | FPG | 0,082 | 0,215 |
| Alloprevotella | insulin | 0,074 | 0,279 |
| Alloprevotella | DBP | 0,068 | 0,314 |
| Alloprevotella | BMI | 0,068 | 0,314 |
| Alloprevotella | waist_circumference | 0,066 | 0,314 |
| Sutterella | triglycerides | 0,066 | 0,314 |
| Negativibacillus | body_weight | 0,063 | 0,330 |
| Prevotella | body_weight | 0,058 | 0,404 |
| Prevotella | insulin | 0,051 | 0,532 |
| Prevotella | waist_circumference | 0,049 | 0,540 |
| Prevotella | DBP | 0,046 | 0,559 |
| Negativibacillus | waist_circumference | 0,044 | 0,572 |
| Alloprevotella | body_weight | 0,043 | 0,573 |
| Prevotella | HOMA-IR | 0,040 | 0,629 |
| Negativibacillus | BMI | 0,038 | 0,629 |
| Sutterella | body_weight | 0,035 | 0,640 |
| Sutterella | waist_circumference | 0,035 | 0,640 |
| Prevotella | FPG | 0,032 | 0,674 |
| Prevotella | BMI | 0,030 | 0,674 |
| Negativibacillus | hba1c | 0,025 | 0,785 |
| Sutterella | tot_cholesterol | 0,023 | 0,785 |
| Prevotella | LDL | 0,021 | 0,796 |
| Sutterella | BMI | 0,018 | 0,835 |
| Negativibacillus | DBP | 0,017 | 0,835 |
| Sutterella | DBP | 0,011 | 0,916 |
| Sutterella | SBP | 0,008 | 0,969 |
| Negativibacillus | tot_cholesterol | 0,006 | 0,969 |
| Negativibacillus | SBP | 0,002 | 0,969 |
| Sutterella | insulin | -0,002 | 0,969 |
| Sutterella | HOMA-IR | -0,002 | 0,969 |
| Prevotella | hba1c | -0,003 | 0,969 |
| Sutterella | hba1c | -0,003 | 0,969 |
| Alloprevotella | LDL | -0,005 | 0,969 |
| Alloprevotella | tot_cholesterol | -0,012 | 0,916 |
| Sutterella | LDL | -0,012 | 0,916 |
| Alloprevotella | triglycerides | -0,021 | 0,796 |
| Prevotella | triglycerides | -0,030 | 0,674 |
| Alloprevotella | SBP | -0,033 | 0,661 |
| Prevotella | tot_cholesterol | -0,035 | 0,640 |
| Negativibacillus | LDL | -0,039 | 0,629 |
| Alloprevotella | HDL | -0,047 | 0,559 |
| Sutterella | HDL | -0,052 | 0,517 |
| Prevotella | SBP | -0,074 | 0,279 |
| Negativibacillus | HDL | -0,081 | 0,215 |
| Prevotella | HDL | -0,090 | 0,188 |
